# Hypertension, Dyslipidemia, and Adhesive Capsulitis: A Bidirectional Two‐Sample Mendelian Randomization Study of the European Population

**DOI:** 10.1155/genr/6618466

**Published:** 2026-05-17

**Authors:** Jianxu Wang, Yijun Xin, Bin Li, Siying Li, Guang Yang

**Affiliations:** ^1^ Department of Joint Surgery, Shandong Provincial Hospital Affiliated to Shandong First Medical University, Jinan, 250021, Shandong, China, sph.com.cn; ^2^ Metabolism and Diseases Research Center, Central Hospital Affiliated to Shandong First Medical University, Jinan, 250013, Shandong, China, sdu.edu.cn

**Keywords:** adhesive capsulitis, causality, dyslipidemia, hypertension, mendelian randomization

## Abstract

**Background:**

Whether hypertension and dyslipidemia are risk factors for adhesive capsulitis (AC) remains controversial. Many observational studies have reported conflicting results. However, observational studies are susceptible to confounding and reverse causation, limiting the ability to establish causality. Therefore, a robust method is needed to clarify these relationships.

**Methods:**

We first conducted a hospital‐based case–control study that included 200 AC patients and 200 controls. Multivariate logistic regression was used to examine the associations of hypertension and dyslipidemia with AC after adjusting for potential confounders. To address the inherent limitations of observational studies, we then performed a two‐sample Mendelian randomization (MR) analysis. We obtained datasets related to essential hypertension, dyslipidemia, and AC from a public genome‐wide association study (GWAS) database. Inverse variance weighted (IVW) served as the primary analysis method. Sensitivity analyses included MR‐PRESSO to detect outliers and pleiotropy and Cochran’s Q test (combined with MR‐Egger and IVW) to assess heterogeneity. The robustness of the findings was evaluated using a leave‐one‐out analysis. Finally, a bidirectional MR analysis was conducted by swapping the exposures and outcomes to test for reverse causality.

**Results:**

In the clinical case–control study, multivariate logistic regression revealed that age (OR: per year: 1.061, 95% CI: 1.034–1.089, *p* < 0.001) and diabetes (OR: 2.153, 95% CI: 1.271–3.646, *p* = 0.004) were independently associated with AC, whereas neither hypertension (OR: 1.406, 95% CI: 0.870–2.274, *p* = 0.164) nor dyslipidemia (OR: 1.760, 95% CI: 0.912–3.395, *p* = 0.092) showed a significant association with AC. Consistent with the observational findings, MR analysis detected no causal effect of hypertension or dyslipidemia on AC. However, reverse MR analysis identified a significant negative causal effect of AC on high‐density lipoprotein (HDL) cholesterol (OR: 0.989, 95% CI: 0.982–0.997, *p* = 0.008) and a positive causal effect of AC on the apolipoprotein B/A1 ratio (OR: 1.018, 95% CI: 1.001–1.034, *p* = 0.033).

**Conclusion:**

Our MR analysis revealed a negative causal effect from AC to HDL cholesterol and a positive causal effect from AC to the apolipoprotein B/A1 ratio. These findings provide evidence for the temporal sequence and reverse causal relationship between AC and dyslipidemia. The convergent results from both clinical and genetic analyses support the robustness of this causal relationship and highlight AC as a potential driver of lipid abnormalities. Therefore, these findings underscore the need for further experimental and mechanistic studies to elucidate the underlying biological mechanisms.

## 1. Introduction

Adhesive capsulitis (AC), also called frozen shoulder, is a common disorder characterized by shoulder pain and loss of passive and active shoulder range of motion. Its prevalence is 2%–5% [1], although estimates range from 0.5% to 10% [2]. AC typically occurs between the ages of 40 and 60 [3] and is more common in women [4].

AC can be divided into primary (spontaneous and unknown etiology) and secondary (caused by surgery, trauma, or other diseases). Several conditions have been considered risk factors for AC, including diabetes [5, 6], hypothyroidism [7–9], cardiac disease [10–12], personality disorder [13], breast cancer treatment [14, 15], and Dupuytren disease [16, 17]. However, because the etiology of AC remains unclear, treatment options are still limited.

Treatment of AC aims to improve functional mobility, reduce pain, and restore proper joint function. Conservative treatment is usually the first‐line approach and includes physical therapy, steroid injections, and anti‐inflammatory medication. For complex and refractory cases, operative treatments are available [18].

Hypertension is a chronic metabolic disease with a high prevalence worldwide. As much as 31.1% of the global adult population was reported to have hypertension in 2010 [19], posing a significant burden to individual and national health services. Hypertension is divided into primary and secondary hypertension, accounting for 90%–95% and 5%–10% of cases, respectively [20].

Although inflammation is known to be a risk factor for hypertension [21], and hypertension may, in turn, be a cause of inflammation [21, 22], the role of hypertension as a risk factor for AC remains debated. Kingston et al. [3] found no significant association between hypertension alone and AC. Conversely, Austin et al. [23] found that patients with AC have a significantly higher rate of antihypertensive drug use, supporting the idea that hypertension may also play a role in the development of AC.

Dyslipidemia is a common metabolic disorder of lipoproteins in the human body, defined as raised plasma concentrations of total cholesterol, low‐density lipoprotein (LDL) cholesterol, or triglycerides, or a low plasma concentration of high‐density lipoprotein (HDL) cholesterol, or a combination thereof [24]. Dyslipidemia is an important risk factor for atherosclerotic cardiovascular diseases such as coronary artery disease and stroke [25], which are the leading causes of death worldwide [26]. Furthermore, abnormal cholesterol is often associated with chronic inflammation throughout the body [27]. However, there is great controversy about the association between dyslipidemia and AC. The prevalence of cholesterol‐lowering medication use in patients with AC has been reported to be similar to that of the general population [23]; a nationwide US study cohort similarly found no association between dyslipidemia and AC. Conversely, Sung et al. [28] concluded that hypercholesterolemia and inflammatory lipoproteinemia have a significant association with primary AC. Wang et al. [29] also identified hyperlipidemia as a risk factor for AC.

Therefore, there is an urgent need for a novel and convincing research method to determine the causal relationship between hypertension, dyslipidemia, and AC. Traditional observational studies are susceptible to confounding factors and reverse causation, which can bias their findings and may explain the conflicting results in the literature. Mendelian randomization (MR), using genetic variants strongly associated with exposure factors from genome‐wide association study (GWAS) data as instrumental variables (IVs), provides a means of estimating causal relationships between exposures and outcomes that satisfy temporal plausibility [30], offering better control for confounding and leveraging large sample sizes. This approach is analogous to a natural randomized controlled trial because alleles are randomly assorted at conception and are typically independent of confounding factors. This methodological framework has been increasingly employed to elucidate links between metabolic and cardiovascular diseases. For instance, a recent two‐sample MR study successfully demonstrated causal relationships between fatty acids and the risk of dilated cardiomyopathy [31], highlighting the value of MR in disentangling complex metabolic pathways. Therefore, building on this approach, we aimed to use single nucleotide polymorphisms (SNPs) that are strongly associated with essential hypertension, LDL cholesterol, HDL cholesterol, apolipoprotein A1 levels, apolipoprotein B levels, triglycerides, and the apolipoprotein B/A1 ratio as IVs to conduct a two‐sample MR analysis examining the causal effect of hypertension and dyslipidemia on AC. Thereafter, SNPs strongly associated with AC were selected for reverse MR to investigate whether there was reverse causality.

## 2. Methods

### 2.1. Study Population

We retrospectively enrolled 200 patients diagnosed with AC at Shandong Provincial Hospital Affiliated to Shandong First Medical University between March 1, 2024, and March 1, 2026. AC was diagnosed based on clinical symptoms and physical examination findings of restricted passive range of motion in the glenohumeral joint. Exclusion criteria included secondary AC (post‐traumatic or postsurgical), rotator cuff tears, shoulder arthritis, and prior shoulder surgery.

Controls were randomly selected from individuals attending the same hospital’s health examination center during the same period, with no history of AC.

### 2.2. Data Collection and Variable Definition

Demographic and clinical data were extracted from electronic medical records, including age, sex, smoking status, and comorbidities (diabetes and thyroid disease). Hypertension was defined as systolic blood pressure ≥ 140 mmHg, diastolic blood pressure ≥ 90 mmHg, or current use of antihypertensive medication. Dyslipidemia was defined by either a clinical diagnosis of hyperlipidemia or current use of lipid‐lowering agents (e.g., statins).

### 2.3. Statistical Analysis

Baseline characteristics were compared between cases and controls using Student’s *t* test for continuous variables and the chi‐square test for categorical variables. Multivariate logistic regression was performed to assess the independent associations of hypertension and dyslipidemia with AC. Covariates included age, sex, smoking, diabetes, and thyroid disease. Results are presented as odds ratios (ORs) with 95% confidence intervals (CIs). Statistical significance was set at *p* < 0.05. All analyses were performed using SPSS 25.0 statistical software.

## 3. Data Summary

Table 1 summarizes the GWAS data sources used for each exposure and outcome, including sample sizes and references.

**TABLE 1:** SNP summary of the exposure and outcome data.

Exposure/outcome	Data source	Year	Population	Sample size	Number of SNPs
Essential hypertension	FinnGen	2021	European	205,684	16,380,443
	UK Biobank	2018	European	463,010	9,851,867

LDL cholesterol	Richardson et al. [32]	2020	European	440,546	12,321,875

HDL cholesterol	Richardson et al. [32]	2020	European	403,943	12,321,875

Triglycerides	Richardson et al. [32]	2020	European	441,016	12,321,875

Apolipoprotein A1	Richardson et al. [32]	2020	European	393,193	12,321,875

Apolipoprotein B	Richardson et al. [32]	2020	European	439,214	12,321,875

Apolipoprotein B/A1 ratio	Richardson et al. [33]	2022	European	115,082	11,590,399

Adhesive capsulitis	Green et al. [34]	2021	European	451,099	15,184,371
	FinnGen	2021	European	170,583	16,380,317

*Note:* SNPs: single nucleotide polymorphisms.

Abbreviations: HDL: high‐density lipoprotein; LDL: low‐density lipoprotein.

### 3.1. SNP Selection

**FIGURE 1:**
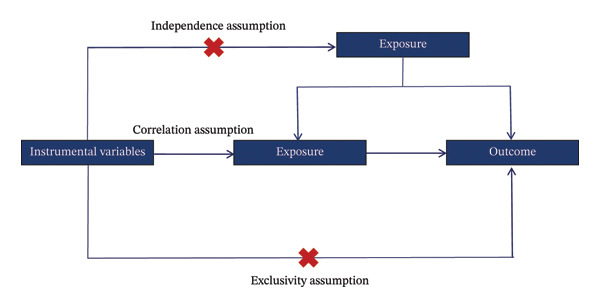
Overview of the design and three key assumptions of the Mendelian randomization.

MR is based on three core assumptions: (i) the IVs are relevant to the exposure factors, (ii) these variables are independent of any confounders that affect the exposure and outcome, and (iii) they influence the outcome solely through the exposure, with no alternative pathways (Figure 1). To satisfy the relevance assumption, we selected IVs that were highly correlated with exposure factors (*p* < 5 × 10^−8^), and then excluded SNPs with *r*2 < 0.01 and kb < 10,000 to eliminate any effects of linkage disequilibrium. We then used the F‐statistic to quantify the strength of IVs, calculated as follows:

(1) F=R2n−212−R,

where *R*2 = [2 × Beta2 × (1 − EAF) × EAF]/[2 × Beta2 × (1 − EAF) × EAF + 2 × SE2 × *N* × (1 − EAF) × EAF]. EAF is the effect allele frequency, and *n* is the GWAS sample size, representing the proportion of exposure variability explained by each IV. SNPs with *F* < 10 were then excluded to avoid weak IVs. Finally, to meet the assumptions of independence and exclusivity, we entered all the previously obtained SNPs into the PhenoScanner (http://www.phenoscanner.medschl.cam.ac.uk/) [35] to retrieve the represented traits and removed SNPs that might be related to confounding factors and may have causal relationship with the outcome of AC, including diabetes, hypothyroidism, obesity, anxiety, depression, ischemic stroke, breast cancer, carpal tunnel syndrome, and Dupuytren disease. The number of SNPs for each step is shown in Supporting Figure 5.

### 3.2. MR Statistical Analysis

We performed bidirectional two‐sample MR to assess causal relationships between hypertension, dyslipidemia, and AC [36]. Analyses were conducted using the TwoSampleMR package (Version 0.5.10) in *R* (Version 4.3.2). SNPs were selected as IVs to represent the exposure factor through rigorous screening for correlation strength with exposure and ensuring the absence of linkage disequilibrium. After extracting the IVs of each exposure factor in the outcome database, we employed the “harmonise_data” function to coordinate exposure and outcome data and removed the palindromic SNPs. Causal effects were estimated primarily by inverse variance weighting (IVW), supplemented by MR‐Egger, weighted median, weighted mode, and simple mode methods. Results are presented as ORs with 95% CIs; statistical significance was set at *p* < 0.05. Cochran’s Q test was used to assess the heterogeneity among SNPs, with *p* < 0.05 considered to be statistically significant, indicating the possibility of heterogeneity with the random effect model IVW (IVW‐RE) used for evaluation; conversely, the fixed effect model IVW (IVW‐FE) was used to evaluate the results. We used the intercept MR‐Egger method to measure horizontal pleiotropy (*p* > 0.05 indicated none), which determined whether the results were available or not (Table 2). MR‐PRESSO was also used to evaluate the stability of the overall data, and SNPs that had a large impact on the results were screened out and removed (Table 3).

**TABLE 2:** Heterogeneity test and pleiotropy test.

Exposure	Outcome	Heterogeneity		Horizontal pleiotropy		
		MR‐Egger	Inverse variance weighted	Egger intercept	Se	*p* value
Essential hypertension	Adhesive capsulitis	0.019	0.015	−0.0394,101	0.03,983,955	0.348,403
Adhesive capsulitis	Essential hypertension	0.054	0.052	−0.000735,268	0.000816,079	0.391,073
LDL cholesterol	Adhesive capsulitis	0.67	0.658	0.007,419,226	0.006,257,444	0.2,387,122
Adhesive capsulitis	LDL cholesterol	0.07	0.079	0.001,729,075	0.002,381,318	0.484
HDL cholesterol	Adhesive capsulitis	0.602	0.574	−0.005,894,643	0.003,727,021	0.1,151,981
Adhesive capsulitis	HDL cholesterol	0.199	0.17	0.002,241,609	0.001,923,617	0.2,709,374
Triglycerides	Adhesive capsulitis	0.046	0.046	0.003,996,531	0.004,187,479	0.3,413,118
Adhesive capsulitis	Triglycerides	0.107	0.127	−0.001,467,753	0.002,267,531	0.5,336,038
Apolipoprotein B	Adhesive capsulitis	0.658	0.663	0.00548,595	0.006,206,408	0.3,788,197
Adhesive capsulitis	Apolipoprotein B	0.037	0.049	0.00119,867	0.002,509,232	0.6,431,309
Apolipoprotein A1	Adhesive capsulitis	0.678	0.654	−0.006,281,473	0.004,254,784	0.1,416,728
Adhesive capsulitis	Apolipoprotein A1	0.065	0.042	0.002,841,529	0.002,255,128	0.2,362,689
Apolipoprotein B/A1 ratio	Adhesive capsulitis	0.105	0.117	−0.006,798,807	0.01,106,048	0.541,853
Adhesive capsulitis	Apolipoprotein B/A1 ratio	0.795	0.746	0.004,183,867	0.003,536,313	0.2,641,273

Abbreviations: HDL: high‐density lipoprotein; LDL: low‐density lipoprotein; MR: Mendelian randomization; Se: standard error.

**TABLE 3:** MR‐PRESSO results.

Exposure	Outcome	MR‐PRESSO global pleiotropy test	
		RSSobs	*p* value
Essential hypertension	Adhesive capsulitis	18.13503	0.114
Adhesive capsulitis	Essential hypertension	23.01381	0.061
LDL cholesterol	Adhesive capsulitis	91.96797	0.739
Adhesive capsulitis	LDL cholesterol	20.73165	0.109
HDL cholesterol	Adhesive capsulitis	220.3048	0.506
Adhesive capsulitis	HDL cholesterol	18.08304	0.205
Triglycerides	Adhesive capsulitis	198.6064	0.057
Adhesive capsulitis	Triglycerides	18.12786	0.133
Apolipoprotein B	Adhesive capsulitis	102.6627	0.611
Adhesive capsulitis	Apolipoprotein B	22.46342	0.087
Apolipoprotein A1	Adhesive capsulitis	176.9073	0.508
Adhesive capsulitis	Apolipoprotein A1	16.61812	0.193
Apolipoprotein B/A1 ratio	Adhesive capsulitis	61.78752	0.084
Adhesive capsulitis	Apolipoprotein B/A1 ratio	8.997,331	0.761

### 3.3. Data Visualization

We used the leave‐one‐out method to determine whether a SNP significantly changed the results. Funnel plots were used to check for heterogeneity across genetic variants. Finally, the results of individual SNPs estimated by the Wald ratio method were expressed in the form of forest plots. Causal estimates (ORs with 95% CIs and *p* values) for exposure and outcome factors were plotted on a single plot to facilitate direct observation (Supporting Figure 1‐4).

## 4. Results

### 4.1. Clinical Associations of Hypertension and Dyslipidemia With AC

The baseline characteristics of the study population are summarized in Table 4. Compared with controls, AC patients were significantly older (58.4 ± 8.2 vs. 54.5 ± 8.3 years, *p* < 0.001) and had a higher proportion of females (56.0% vs. 51.0%, *p* = 0.316), although this difference did not reach statistical significance. Diabetes was more prevalent in AC (27.0% vs. 14.0%, *p* = 0.001). No significant differences were observed for smoking, thyroid disease, hypertension, or dyslipidemia between the two groups.

**TABLE 4:** Baseline characteristics of the study population.

Characteristic	Controls (*n* = 200)	AC cases (*n* = 200)	*p* value
Age, years	54.5 ± 8.3	58.4 ± 8.2	< 0.001
Female, *n* (%)	102 (51.0%)	112 (56.0%)	0.316
Smoking, *n* (%)	56 (28%)	60 (30%)	0.659
Diabetes, *n* (%)	28 (14%)	54 (27%)	0.001
Thyroid disease, *n* (%)	10 (5%)	14 (7%)	0.400
Hypertension, *n* (%)	46 (23%)	54 (27%)	0.356
Dyslipidemia, *n* (%)	20 (10%)	28 (14%)	0.218

Multivariate logistic regression analysis was performed to assess the independent associations of hypertension and dyslipidemia with AC, with results presented in Table 5. Age (OR per year: 1.061, 95% CI: 1.034–1.089, *p* < 0.001) and diabetes (OR: 2.153, 95% CI: 1.271–3.646, *p* = 0.004) were independently associated with AC. Hypertension (OR: 1.406, 95% CI: 0.870–2.274, *p* = 0.164) and dyslipidemia (OR: 1.760, 95% CI: 0.912–3.395, *p* = 0.092) were not associated with AC.

**TABLE 5:** Results of multivariate logistic regression analysis.

Variable	OR	95% CI	*p* value
Age (per year)	1.061	1.034–1.089	< 0.001
Sex (female vs. male)	1.248	0.820–1.901	0.302
Smoking (yes vs. no)	1.126	0.715–1.775	0.609
Diabetes (yes vs. no)	2.153	1.271–3.646	0.004
Thyroid disease (yes vs. no)	1.389	0.578–3.337	0.462
Hypertension (yes vs. no)	1.406	0.870–2.274	0.164
Dyslipidemia (yes vs. no)	1.760	0.912–3.395	0.092

### 4.2. Essential Hypertension and AC

To clearly distinguish between the two directions of investigation, we present forward MR results (examining causal effects of essential hypertension or dyslipidemia on AC) followed by reverse MR results (examining causal effects of AC on essential hypertension or dyslipidemia).

For essential hypertension, we selected 14 SNPs as IVs after rigorous screening. The F‐statistics for all SNPs were above 10, indicating no weak instrument bias (Supporting Table 1). The primary IVW‐RE analysis revealed no causal effect of essential hypertension on the risk of AC (OR: 0.904, 95% CI: 0.739–1.106, *p* = 0.326). No outliers were found by the MR‐PRESSO test, and the leave‐one‐out method demonstrated that our results were not affected by a single SNP.

In the reverse MR analysis, we treated AC as the exposure factor. Initially, only one SNP met the significance threshold (*p* < 5 × 10^−8^) under the relevance assumption, so we relaxed the threshold to 5 × 10^−6^ and obtained 12 SNPs. All of these SNPs passed the weak instrument test (F‐statistic > 10) (Supporting Table 8). The IVW‐FE analysis did not support a causal effect of AC on essential hypertension (OR: 0.999, 95% CI: 0.997–1.002, *p* = 0.850). No outliers were found by the MR‐PRESSO test, and the leave‐one‐out method also showed that our results are stable (Table 6).

**TABLE 6:** MR analysis results of essential hypertension and adhesive capsulitis.

Exposure	SNPs	Outcome	Methods	*p* value	OR	95% CI
Essential hypertension	14	Adhesive capsulitis	IVW‐RE	0.326	0.904	0.739–1.106
			MR‐Egger	0.477	1.4	0.575–3.41
			Weighted median	0.353	0.905	0.732–1.118
			Weighted mode	0.503	0.87	0.589–1.287
			Simple mode	0.581	0.89	0.597–1.328

Adhesive capsulitis	12	Essential hypertension	IVW‐FE	0.85	0.999	0.997–1.002
			MR‐Egger	0.471	1.003	0.995–1.012
			Weighted median	0.605	0.999	0.995–1.003
			Weighted mode	0.396	0.995	0.985–1.006
			Simple mode	0.321	0.996	0.988–1.004

*Note:* SNPs: single nucleotide polymorphisms.

Abbreviations: CI, confidence interval; IVW, inverse variance weighted; OR, odds ratio.

### 4.3. Dyslipidemia and AC

We systematically investigated the causal role of seven lipid‐related traits on AC. LDL cholesterol (OR: 0.906, 95% CI: 0.701–1.171, *p* = 0.451), HDL cholesterol (OR: 1.158, 95% CI: 0.980–1.368, *p* = 0.084), triglycerides (OR: 1.008, 95% CI: 0.823–1.235, *p* = 0.939), apolipoprotein B (OR: 1.102, 95% CI: 0.859–1.414, *p* = 0.445), apolipoprotein A1 (OR: 1.119, 95% CI: 0.929–1.349, *p* = 0.235), and the apolipoprotein B/A1 ratio (OR: 0.835, 95% CI: 0.681–1.025, *p* = 0.084) showed no causal effect on AC. SNP details are presented in Supporting Tables 2–7.

In contrast to the forward direction, the reverse MR analysis revealed significant causal effects of AC on specific lipid traits. AC showed a significant negative causal effect on HDL cholesterol (OR: 0.989, 95% CI: 0.982–0.997, *p* = 0.008) and a positive causal effect on the apolipoprotein B/A1 ratio (OR: 1.018, 95% CI: 1.001–1.034, *p* = 0.033) using the IVW method. No causal effects of AC were observed on LDL cholesterol (OR: 1.003, 95% CI: 0.994–1.011, *p* = 0.547), triglycerides (OR: 1.004, 95% CI: 0.996–1.012, *p* = 0.349), apolipoprotein B (OR: 1.002, 95% CI: 0.992–1.014, *p* = 0.614), or apolipoprotein A1 (OR: 0.993, 95% CI: 0.984–1.004, *p* = 0.254) (Table 7).

**TABLE 7:** MR analysis results of dyslipidemia and adhesive capsulitis.

Exposure	SNPs	Outcome	Methods	*p* value	OR	95% CI
LDL cholesterol	111	Adhesive capsulitis	IVW‐FE	0.451	0.906	0.701–1.171
			MR‐Egger	0.163	0.711	0.442–1.144
			Weighted median	0.083	0.679	0.439–1.052
			Weighted mode	0.672	0.698	0.132–3.677
			Simple mode	0.359	0.679	0.298–1.547

Adhesive capsulitis	12	LDL cholesterol	IVW‐FE	0.547	1.003	0.994–1.011
			MR‐Egger	0.653	0.993	0.969–1.02
			Weighted median	0.954	1	0.989–1.012
			Weighted mode	0.93	0.999	0.976–1.022
			Simple mode	0.6	0.995	0.978–1.013

HDL cholesterol	248	Adhesive capsulitis	IVW‐FE	0.084	1.158	0.98–1.368
			MR‐Egger	0.022	1.379	1.049–1.813
			Weighted median	0.215	1.192	0.903–1.574
			Weighted mode	0.738	1.332	0.248–7.14
			Simple mode	0.883	1.057	0.508–2.196

Adhesive capsulitis	12	HDL cholesterol	**IVW-FE**	**0.008**	**0.989**	**0.982–0.997**
			MR‐Egger	0.07	0.979	0.959–0.999
			Weighted median	0.954	1	0.989–1.012
			Weighted mode	0.512	0.992	0.969–1.015
			Simple mode	0.549	0.993	0.972–1.015

Triglycerides	187	Adhesive capsulitis	IVW‐RE	0.939	1.008	0.823–1.235
			MR‐Egger	0.529	0.911	0.68–1.219
			Weighted median	0.936	1.015	0.714–1.442
			Weighted mode	0.973	1.049	0.07–15.772
			Simple mode	0.102	2.009	0.874–4.618

Adhesive capsulitis	12	Triglycerides	IVW‐FE	0.349	1.004	0.996–1.012
			MR‐Egger	0.39	1.011	0.987–1.035
			Weighted median	0.313	1.006	0.994–1.018
			Weighted mode	0.62	1.006	0.984–1.027
			Simple mode	0.602	1.005	0.987–1.024

Apolipoprotein B	115	Adhesive capsulitis	IVW‐FE	0.445	1.102	0.859–1.414
			MR‐Egger	0.767	0.936	0.602–1.454
			Weighted median	0.972	1.008	0.652–1.558
			Weighted mode	0.993	0.992	0.161–6.122
			Simple mode	0.513	1.428	0.493–4.132

Adhesive capsulitis	12	Apolipoprotein B	IVW‐RE	0.614	1.002	0.992–1.014
			MR‐Egger	0.825	0.997	0.971–1.024
			Weighted median	0.75	1.002	0.991–1.013
			Weighted mode	0.777	1.003	0.984–1.023
			Simple mode	0.786	0.998	0.980–1.015

Apolipoprotein A1	200	Adhesive capsulitis	IVW‐FE	0.235	1.119	0.929–1.349
			MR‐Egger	0.06	1.353	0.989–1.849
			Weighted median	0.106	1.293	0.946–1.766
			Weighted mode	0.752	1.341	0.218–8.253
			Simple mode	0.545	1.575	0.363–6.838

Adhesive capsulitis	12	Apolipoprotein A1	IVW‐RE	0.254	0.993	0.984–1.004
			MR‐Egger	0.133	0.98	0.957–1.004
			Weighted median	0.325	0.994	0.983–1.006
			Weighted mode	0.503	0.991	0.967–1.016
			Simple mode	0.593	0.994	0.972–1.016

Apolipoprotein B/A1 ratio	51	Adhesive capsulitis	IVW‐FE	0.084	0.835	0.681–1.025
			MR‐Egger	0.769	0.937	0.607–1.444
			Weighted median	0.561	0.906	0.65–1.264
			Weighted mode	0.983	0.986	0.27–3.6
			Simple mode	0.871	0.918	0.33–2.555

Adhesive capsulitis	12	Apolipoprotein B/A1 ratio	**IVW-FE**	**0.033**	**1.018**	**1.001–1.034**
			MR‐Egger	0.869	0.997	0.96–1.035
			Weighted median	0.256	1.012	0.991–1.033
			Weighted mode	0.725	1.007	0.97–1.045
			Simple mode	0.586	1.010	0.975–1.047

*Note:* SNPs: single nucleotide polymorphisms; ORs: odds ratios; CIs: confidence intervals. The values shown in bold indicate statistically significant differences.

## 5. Discussion

The pathological conditions characterized by abdominal obesity, insulin resistance, hypertension, and hyperlipidemia are collectively known as the metabolic syndrome. As infectious diseases are increasingly controlled, the incidence of this chronic systemic condition has been gradually increasing, posing a significant threat to public health. Metabolic syndrome is approximately three times more common than diabetes [37].

AC is a common disease with a poorly understood etiology. Although hyperglycemia [34, 38] and obesity [14, 39] have been established as risk factors, whether hypertension and dyslipidemia also contribute to AC remains controversial. To address this uncertainty, we conducted a hospital‐based case–control study followed by a bidirectional two‐sample MR analysis to systematically evaluate the associations and causal relationships between hypertension, dyslipidemia, and AC.

In our observational study, age and diabetes were independently associated with AC, consistent with established risk factors for this condition. After multivariable adjustment, however, neither hypertension nor dyslipidemia showed a significant independent association with AC.

To overcome the inherent limitations of observational research—particularly residual confounding and reverse causation—we next performed a two‐sample MR analysis.

In assessing the causal relationship between hypertension and AC, we focused on essential hypertension, as it accounts for the majority of cases and represents a persistent pathological state distinct from secondary hypertension, which typically resolves upon treatment of the underlying condition. Kingston et al. [3] observed that hypertension had a 55.3% incidence in people with AC, but a 61.3% incidence in the control group. Patients with AC were 7% less likely to develop hypertension compared to the control group. They therefore concluded that hypertension alone was not significantly associated with AC. Similarly, Mello et al. [40] found that during the COVID‐19 pandemic, although AC patients had a higher prevalence of hypertension as a comorbidity, the difference was not statistically significant compared to non‐AC individuals with hypertension.

However, Austin et al. [23] found that, in their group of AC, the overall rate of use of hypertension medications among patients ≥ 18 years of age was 33.1%, significantly higher than the previously observed prevalence of 21.6% in the NHANES study. Additionally, after AC patients were divided into traumatic and idiopathic subgroups, the utilization rate of antihypertensive drugs in each group was also notably higher than that observed in the general population. However, the study could not identify diet‐controlled or unmedicated hypertensive patients. Another limitation was the small sample size, which raised the possibility of Type II errors. Diabetes and hypertension have a high comorbidity rate [41], and there may be a causal relationship between the two [42, 43]. Consequently, it is difficult to ascertain from observational studies whether hypertension in AC patients is driven by diabetes or abnormal blood glucose or represents an independent risk factor. Our findings are consistent with several observational studies. Although hypertension is a chronic condition with systemic proinflammatory effects, our MR results indicate that it does not exert a causal effect on AC. The frequent co‐occurrence of hypertension in AC patients observed in clinical studies likely reflects shared underlying factors, rather than a direct causal relationship. Further mechanistic studies are needed to elucidate the biological pathways underlying this clinical comorbidity.

There is a considerable debate as to whether dyslipidemia is an independent risk factor for AC. Sung et al. [28] concluded that hypercholesterolemia, especially high LDL cholesterol and high non‐HDL cholesterol, is significantly associated with primary AC. Although they excluded patients with diabetes and thyroid abnormalities, significant differences other than dyslipidemia may still exist between the control and case groups due to the inability to fully control for confounding factors. Moreover, the large size of the control group increased the likelihood of detecting statistically significant differences, even if clinically modest [44]. As stated, “further research is needed to assess whether nonoptimal lipid levels are a cause, a related cofactor, or a consequence of primary periarthritis of the shoulder.” Similarly, Park et al. [45] reported an association between dyslipidemia and AC using univariate and multivariate logistic regression, though the study shared similar methodological limitations as those of Sung et al. In support of the association between dyslipidemia and AC, Wang et al. [29] found that patients with hyperlipidemia had 1.5 times the risk of developing AC compared with healthy controls, but statin use in patients with hyperlipidemia did not prevent AC.

Many researchers oppose the association between dyslipidemia and AC. Austin et al. [23] found that the overall rate of AC patients using cholesterol‐lowering drugs was 20.6%, similar to the 16.1% observed in the national level of NHANES study. Additionally, in patients with primary AC, the utilization rate of lipid‐lowering medications was 26.7%, consistent with the national level. Therefore, dyslipidemia did not appear to be associated with AC. Moreover, Milgrom et al. [46] found no significant differences in the frequency of hypercholesterolemia between a frozen shoulder group and control groups. As confirmed in our results, LDL cholesterol, triglycerides, apolipoprotein A1, apolipoprotein B, and the apolipoprotein B/A1 ratio showed no causal effect on AC, nor did HDL cholesterol. However, the reverse MR analysis revealed a significant causal effect of AC on lipid metabolism: AC exerted a negative causal effect on HDL cholesterol and a positive causal effect on the apolipoprotein B/A1 ratio.

These findings have important implications for interpreting previous observational studies. While several studies have reported an association between dyslipidemia and AC, our results suggest that this observed association is likely driven by AC‐induced changes in lipid profiles, rather than dyslipidemia predisposing to AC. The inability of lipid‐lowering medications to prevent AC further supports this interpretation. Thus, dyslipidemia appears to be a consequence rather than a cause of AC. This temporal sequence—AC preceding and causing alterations in HDL cholesterol and the apolipoprotein B/A1 ratio—provides a novel perspective on the relationship between these conditions.

The reverse causal relationships identified in our study—namely, that AC predisposes to reduced HDL cholesterol and an elevated apolipoprotein B/A1 ratio—raise intriguing questions about the underlying biological mechanisms. One plausible explanation is the role of chronic low‐grade inflammation. AC is fundamentally a fibroinflammatory condition, characterized by synovial inflammation and capsular fibrosis involving cytokines such as interleukin‐6 (IL‐6) and tumor necrosis factor‐alpha (TNF‐α) [47]. Emerging evidence suggests that such inflammatory states can systemically influence lipid metabolism [48]. For instance, chronic inflammation has been shown to alter lipoprotein function and composition, leading to lower HDL cholesterol levels and a shift toward a more proatherogenic lipid profile [49].

Notably, recent research has demonstrated that inflammatory markers are associated with an increased risk of hypertension in autoimmune conditions such as primary Sjögren’s syndrome (pSS) [50]. Although pSS and AC differ in their primary pathology, both involve a sustained inflammatory burden. This parallel suggests that the local inflammatory process in AC may contribute to systemic inflammatory changes. This AC‐induced inflammation could, in turn, influence both blood pressure regulation and lipid metabolism. Therefore, while hypertension and dyslipidemia do not predispose to AC, the inflammatory milieu associated with AC may help explain the observed clustering of hypertension and dyslipidemia in AC patients.

The observed causal effects of AC on HDL cholesterol and the apolipoprotein B/A1 ratio have potential clinical implications that merit consideration. First, these lipid alterations may serve as early biomarkers for subclinical or prodromal AC. In clinical practice, patients often present with established AC when pain and range of motion limitations have developed, potentially delaying optimal intervention. Routine lipid profiles, which are widely available and cost‐effective, could theoretically identify individuals at elevated risk for AC before overt symptoms emerge. For instance, an unexplained decline in HDL cholesterol or an elevated apolipoprotein B/A1 ratio in a patient without traditional metabolic risk factors might prompt clinicians to inquire about shoulder symptoms or perform a targeted physical examination, facilitating earlier diagnosis.

Second, these findings raise the possibility of surrogate indicators for monitoring AC progression or treatment response. While the current assessment of AC relies primarily on clinical examination and patient‐reported outcomes, objective biomarkers are lacking. Longitudinal studies could investigate whether normalization of HDL cholesterol or the apolipoprotein B/A1 ratio accompanies successful treatment of AC, potentially providing a measurable parameter for therapeutic monitoring.

Third, the identification of AC‐induced dyslipidemia suggests that preventive strategies targeting cardiovascular health may be warranted in AC patients. Even if these lipid changes are modest at the individual level, they could contribute to long‐term cardiovascular risk accumulation, particularly in patients with recurrent or bilateral AC. Clinicians managing AC should be aware of this potential metabolic consequence and consider periodic cardiovascular risk assessment in these patients.

However, it is important to emphasize that these implications are currently hypothetical and require validation through prospective cohort studies. Future research should examine whether lipid abnormalities correlate with AC severity or duration and whether they normalize with successful AC treatment. If confirmed, these lipid biomarkers could offer a simple, noninvasive tool for AC risk stratification and monitoring.

There are several limitations to our study. First, the clinical case–control study has inherent limitations, including potential selection bias, residual confounding, and the inability to establish temporality due to its retrospective design. Second, while the use of European‐ancestry GWAS data minimizes bias due to population stratification, it also limits the generalizability of our findings to non‐European populations. Epidemiological studies have revealed population‐specific patterns in metabolic risk factors and the prevalence of hypertension and dyslipidemia. For instance, recent research in patients with primary Sjögren’s syndrome (pSS) has demonstrated that the relationship between metabolic factors and hypertension may vary across ethnic groups [51]. Therefore, future MR studies using GWAS data from diverse populations—such as East Asian, African, or Hispanic cohorts—are warranted to validate our findings and assess the consistency of these causal relationships across ethnicities. Third, due to the inherent limitations of the open GWAS data, our study could not be stratified by sex and age to examine potential causal effects on hypertension and dyslipidemia. Fourth, to increase the number of SNPs in reverse MR, we adjusted the *p* value to 5e‐6 when selecting IVs for AC, and there were no weak IVs. Fifth, even though the datasets we used for analysis originated from different research alliances, we admit that there may be a very slight degree of overlap. Future replication studies using completely independent datasets will help to further verify our findings.

Despite these limitations, our study has several innovative aspects. For the first time, we applied MR analysis to examine bidirectional causality between hypertension, dyslipidemia, and AC, complementing observational findings. Moreover, we identified that AC exerts causal effects on HDL cholesterol and the apolipoprotein B/A1 ratio—findings not previously reported. The integration of both clinical and genetic evidence further strengthens the validity of these novel findings.

## 6. Conclusion

In this study, we integrated a hospital‐based case–control study with bidirectional two‐sample MR to investigate the associations and causal relationships between hypertension, dyslipidemia, and AC. The case–control study revealed no independent associations of hypertension or dyslipidemia with AC, and MR analysis consistently found no causal effects of these factors on AC. However, reverse MR analysis identified significant causal effects of AC on lipid metabolism: a negative effect on HDL cholesterol and a positive effect on the apolipoprotein B/A1 ratio. These bidirectional MR findings, consistent with the observational results, provide robust evidence that hypertension and dyslipidemia do not causally influence AC, whereas AC itself may induce lipid disturbances. Our study thus offers novel insights into AC etiology and underscores the need for further mechanistic studies to elucidate the biological pathways underlying these reverse causal relationships.

**Nomenclature**
AC: adhesive capsulitis
GWAS: genome‐wide association studies
IVW: inverse variance weighted
HDL: high‐density lipoprotein
LDL: low‐density lipoprotein
MR: Mendelian randomization
IVs: instrumental variables
SNPs: single nucleotide polymorphisms
ORs: odds ratios
CIs: confidence intervals.

## Author Contributions

Jianxu Wang: writing–original draft, visualization, validation, methodology, investigation, and data curation. Yijun Xin: writing–original draft, visualization, validation, and methodology. Bin Li: writing–original draft, investigation, and data curation. Siying Li: writing–review and editing, and validation. Guang Yang: writing–review and editing, supervision, project administration, and funding acquisition.

## Funding

This study did not receive any specific funding.

## Ethics Statement

This study was conducted in accordance with the Declaration of Helsinki. The clinical data were collected retrospectively from routine medical records and analyzed anonymously; therefore, informed consent was not required. The publicly available GWAS data used in the Mendelian randomization analysis did not require additional ethical approval.

## Conflicts of Interest

The authors declare no conflicts of interest.

## Supporting Information

Additional supporting information can be found online in the Supporting Information section.

## Supporting information


**Supporting Information 1** Supporting 1. Supporting Figure 1. Funnel plot of the MR results between exposures and outcome.

**Supporting Information 2** Supporting 2. Supporting Figure 2. Scatter plot of the MR results between the exposures and outcome.

**Supporting Information 3** Supporting 3. Supporting Figure 3. Leave‐one‐out analysis of the MR results between the exposures and outcome.

**Supporting Information 4** Supporting 4. Supporting Figure 4. Forest plot of the MR results between the exposures and outcome.

**Supporting Information 5** Supporting 5. Supporting Figure 5. The number of SNPs filtered at each step.

**Supporting Information 6** Supporting 6. Supporting Table 1. Instrumental variables of essential hypertension.

**Supporting Information 7** Supporting 7. Supporting Table 2. Instrumental variables of LDL cholesterol.

**Supporting Information 8** Supporting 8. Supporting Table 3. Instrumental variables of HDL cholesterol.

**Supporting Information 9** Supporting 9. Supporting Table 4. Instrumental variables of triglycerides.

**Supporting Information 10** Supporting 10. Supporting Table 5. Instrumental variables of apolipoprotein B.

**Supporting Information 11** Supporting 11. Supporting Table 6. Instrumental variables of apolipoprotein A1.

**Supporting Information 12** Supporting 12. Supporting Table 7. Instrumental variables of the apolipoprotein B/A1 ratio.

**Supporting Information 13** Supporting 13. Supporting Table 8. Instrumental variables of AC.

**Supporting Information 14** Graphical abstract: This graphical abstract summarizes a bidirectional two‐sample Mendelian randomization study investigating the relationship between hypertension, dyslipidemia, and adhesive capsulitis (AC) in a European population. Multivariable logistic regression identified age and diabetes as significant risk factors for AC, while hypertension and dyslipidemia were not significantly associated. Forward Mendelian randomization analysis further showed no causal effect of hypertension or dyslipidemia on AC, whereas reverse MR suggested that AC may have a negative causal effect on HDL cholesterol and a positive causal effect on the apolipoprotein B/A1 ratio.

## Data Availability

The clinical data supporting the findings of the case–control study are not publicly available due to patient privacy and confidentiality restrictions but are available from the corresponding author upon reasonable request. The GWAS summary datasets used for the Mendelian randomization analysis are publicly available from the IEU OpenGWAS project (https://gwas.mrcieu.ac.uk/) and the FinnGen database (http://www.finngen.fi).
