# Supplementary material for: Hypertension, Dyslipidemia, and Adhesive Capsulitis: A Bidirectional Two‐Sample Mendelian Randomization Study of the European Population
Source: Genet Res (Camb). 2026 May 17;2026:6618466. doi: 10.1155/genr/6618466 (PMC13180687; doi:10.1155/genr/6618466)
Supplement: Supplementary file 2 — Supporting Information 2 Supporting 2. Supporting Figure 2. Scatter plot of the MR results between the exposures and outcome. [file GENR-2026-6618466-s012.doc]

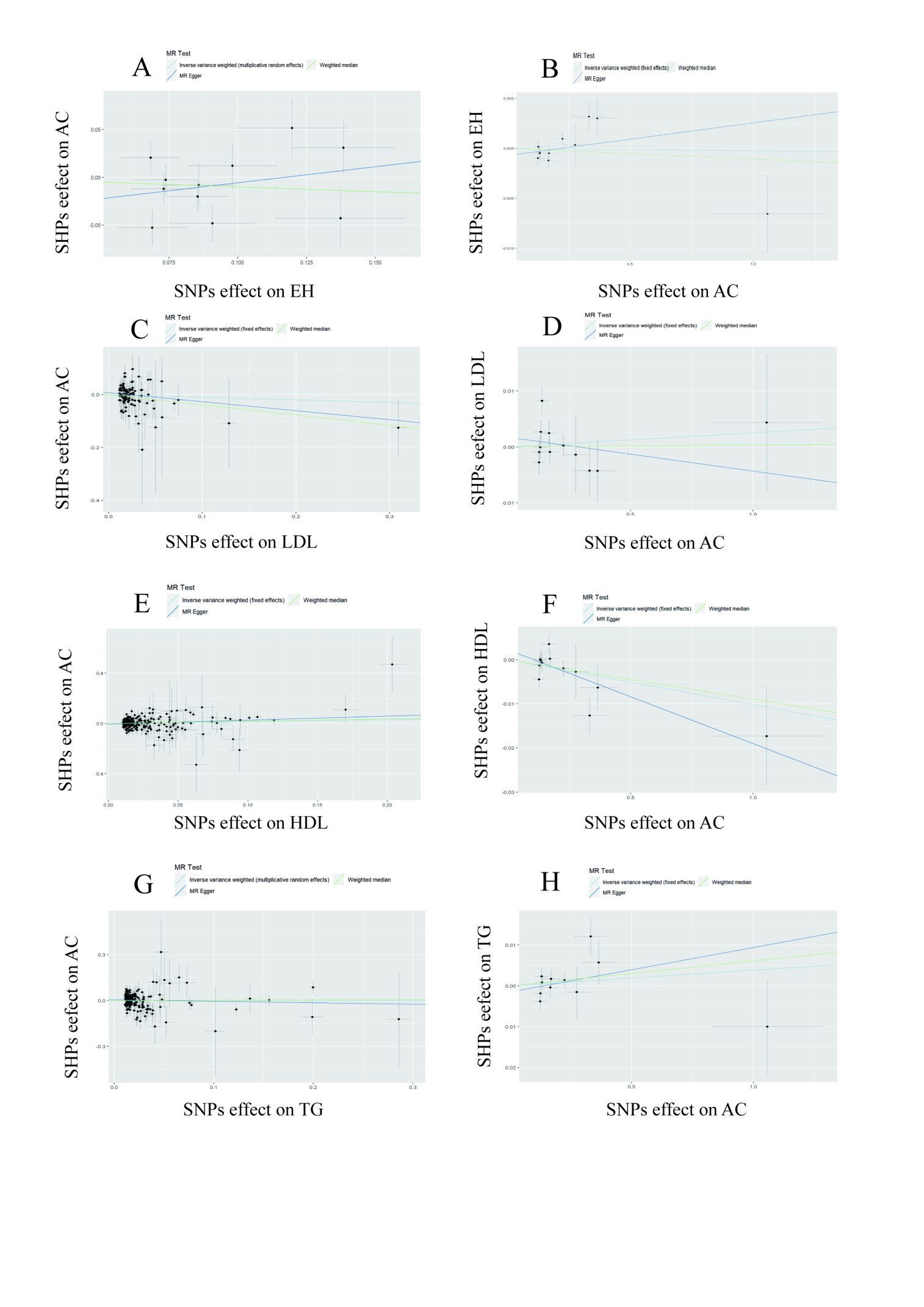
**Supplementary Figure 2** Scatter plot of the MR results between exposures and outcome

**
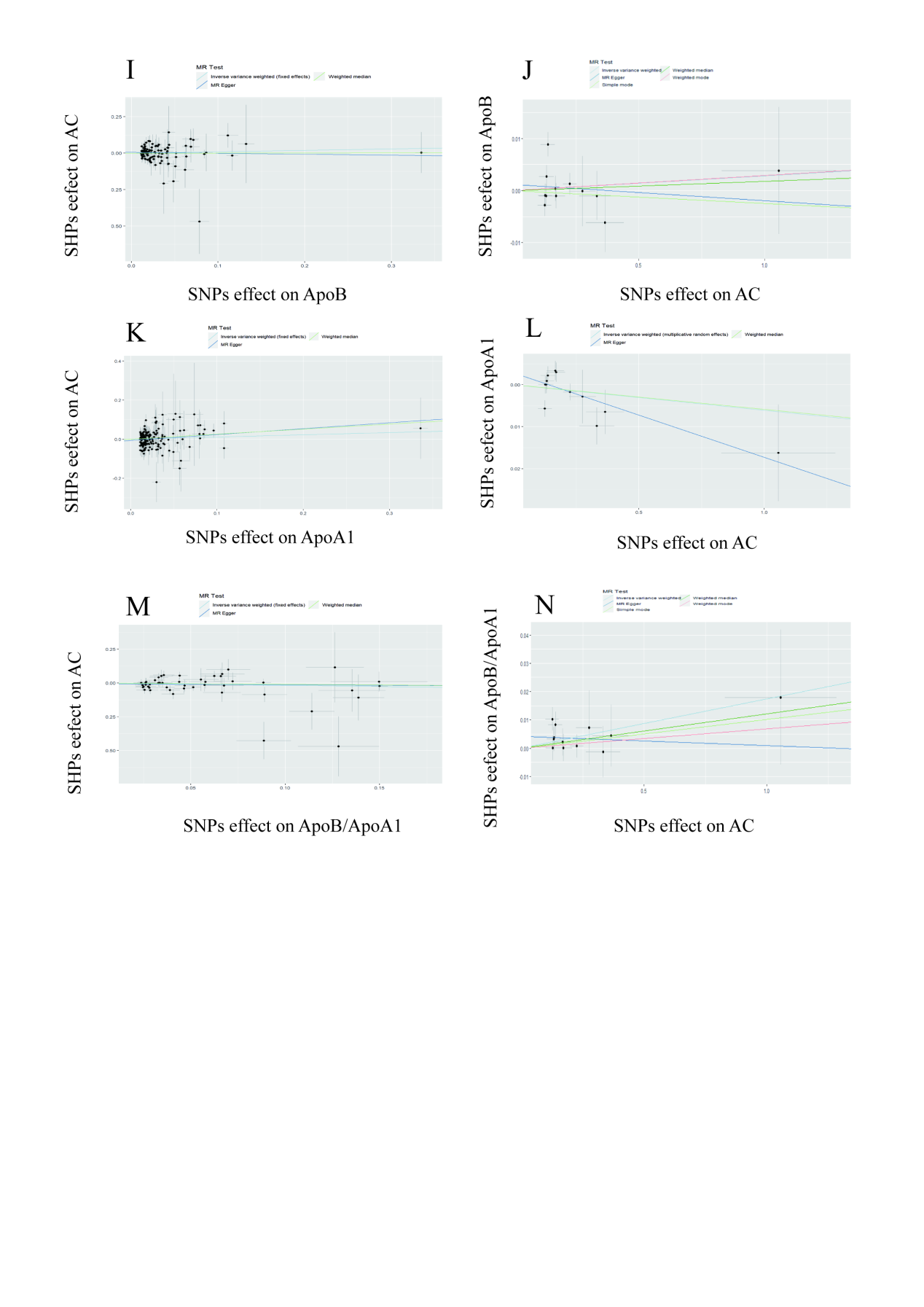
**

MR: mendelian randonmization; EH: essential hypertension; AC: adhesive capsulitis; LDL: low density lipoprotein; HDL: high density lipoprotein; TG: triglyceride; ApoB: apolipoprotein B; ApoA1: apolipoprotein A1; ApoB/ApoA1:apolipoprotein B/apolipoprotein A1 ratio
