# Supplementary material for: Hypertension, Dyslipidemia, and Adhesive Capsulitis: A Bidirectional Two‐Sample Mendelian Randomization Study of the European Population
Source: Genet Res (Camb). 2026 May 17;2026:6618466. doi: 10.1155/genr/6618466 (PMC13180687; doi:10.1155/genr/6618466)
Supplement: Supplementary file 3 — Supporting Information 3 Supporting 3. Supporting Figure 3. Leave‐one‐out analysis of the MR results between the exposures and outcome. [file GENR-2026-6618466-s014.doc]

**Supplementary Figure 3** Leave one out analysis of the MR results between exposures and outcome
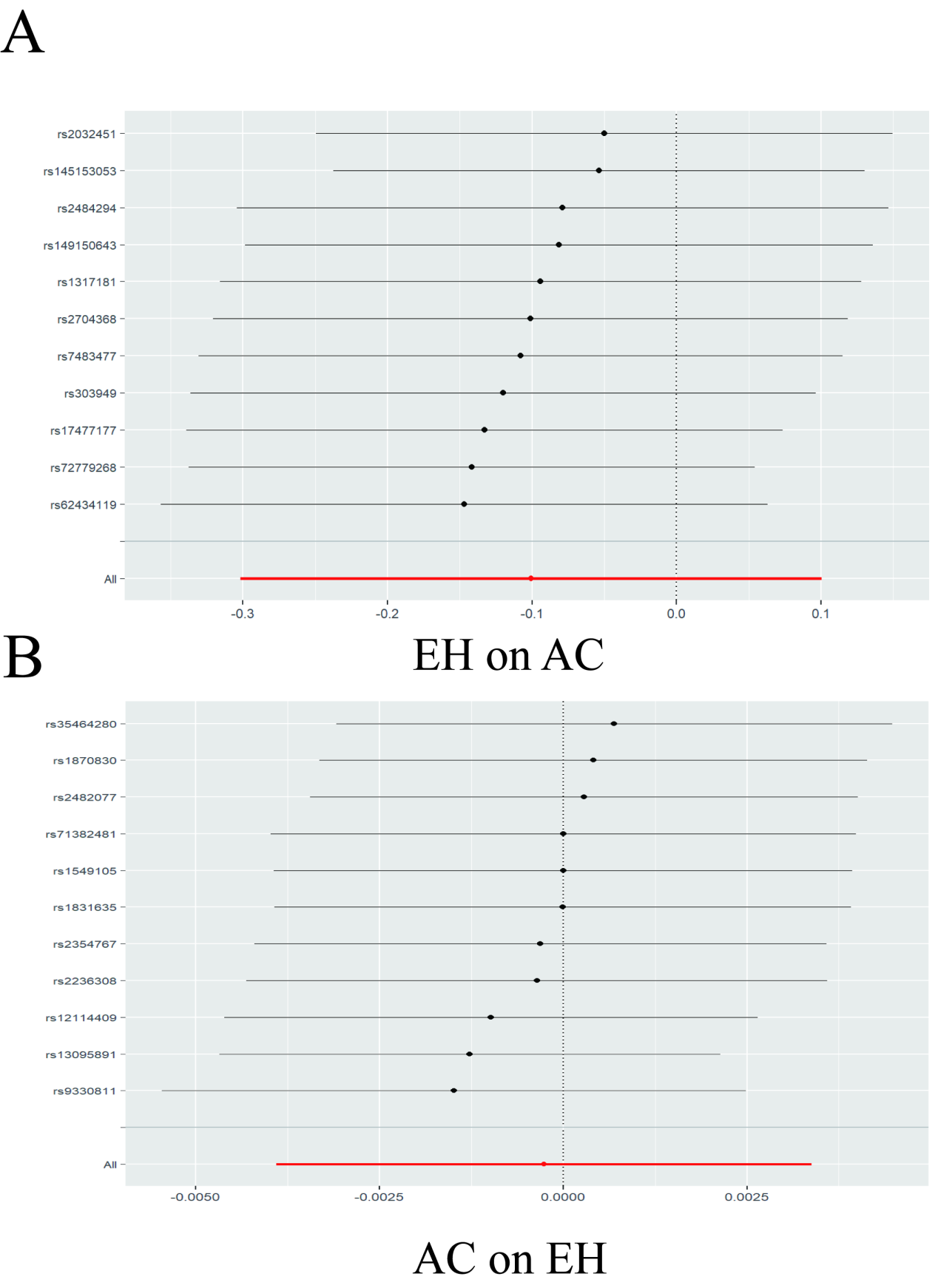


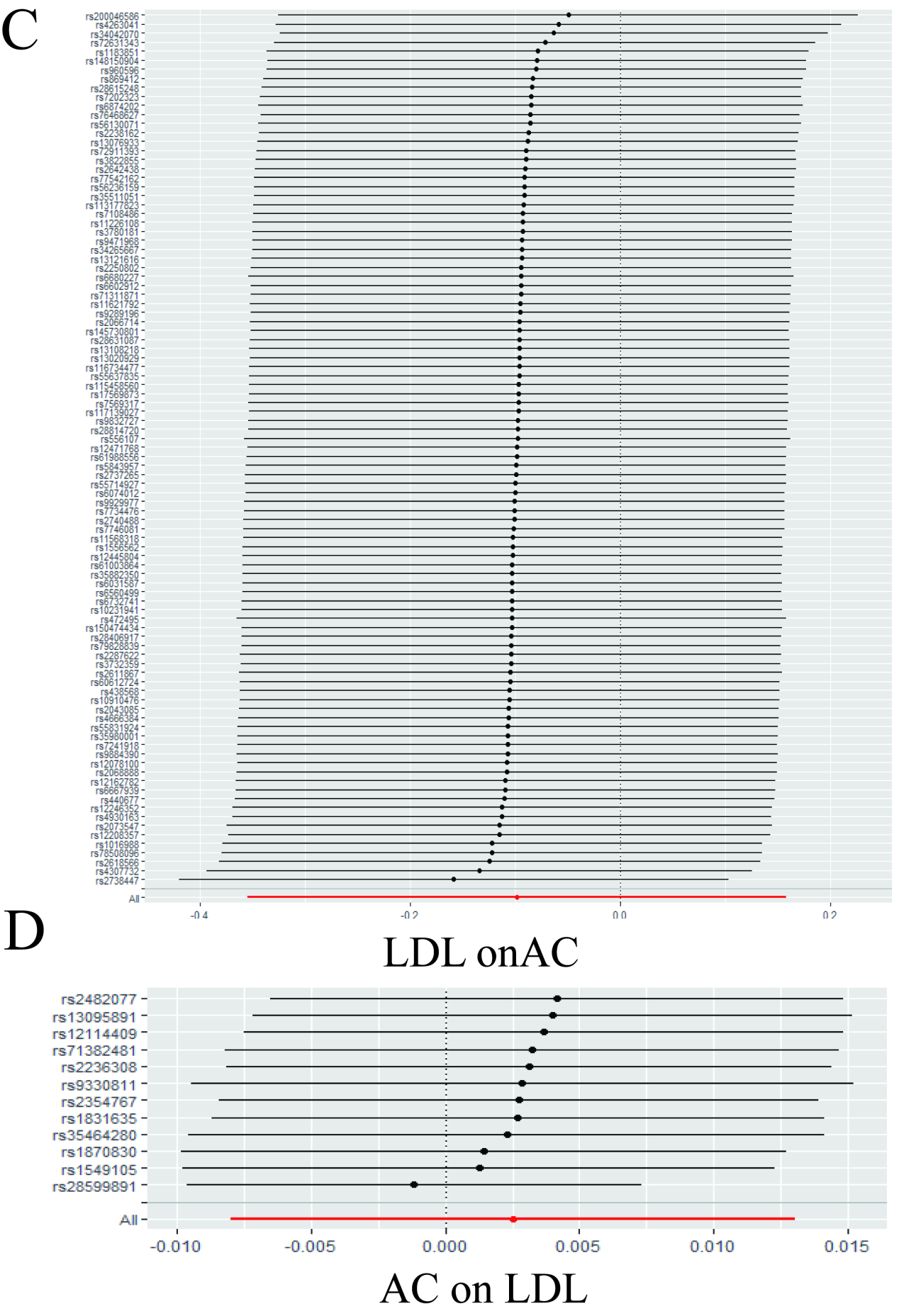


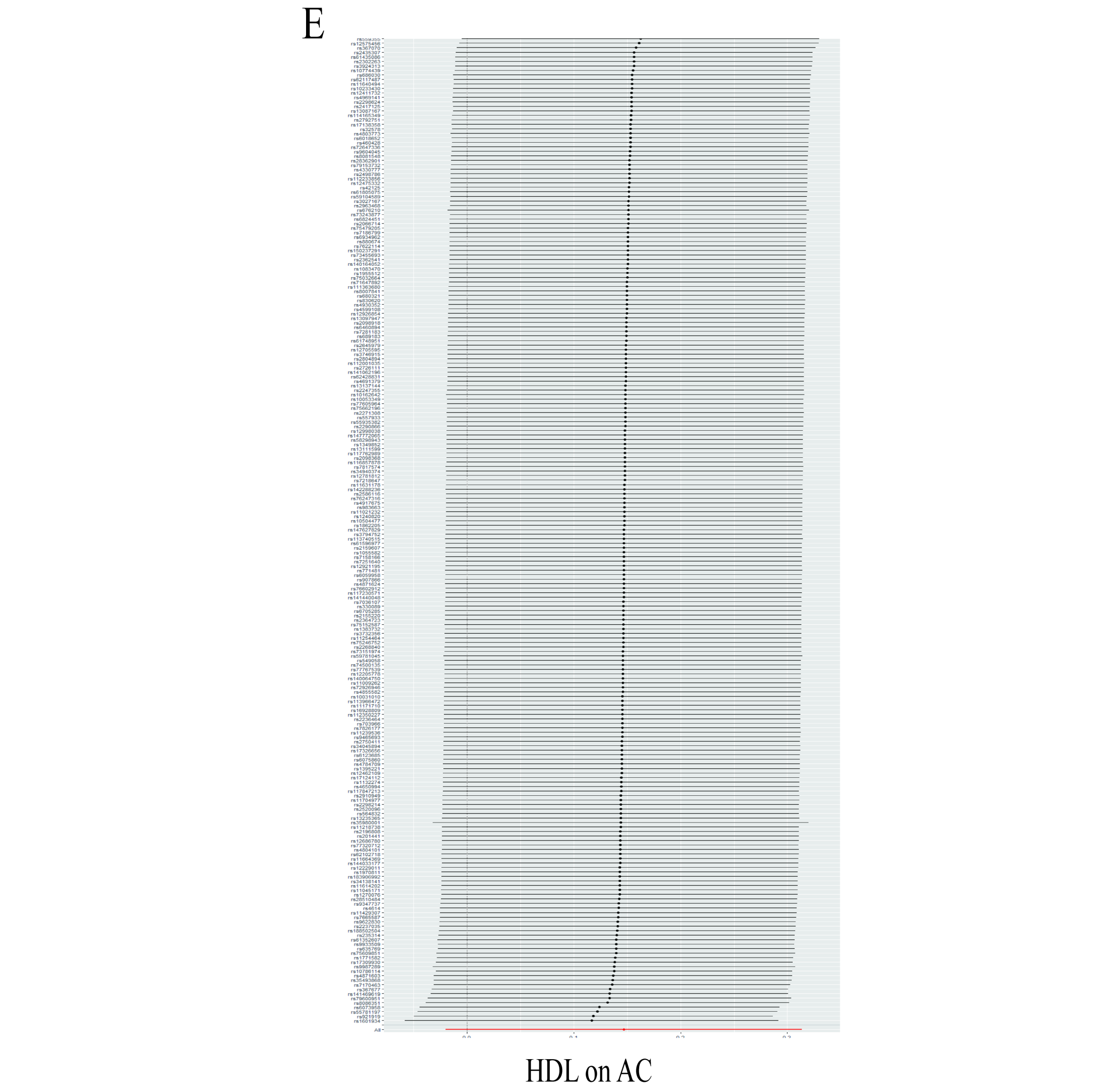


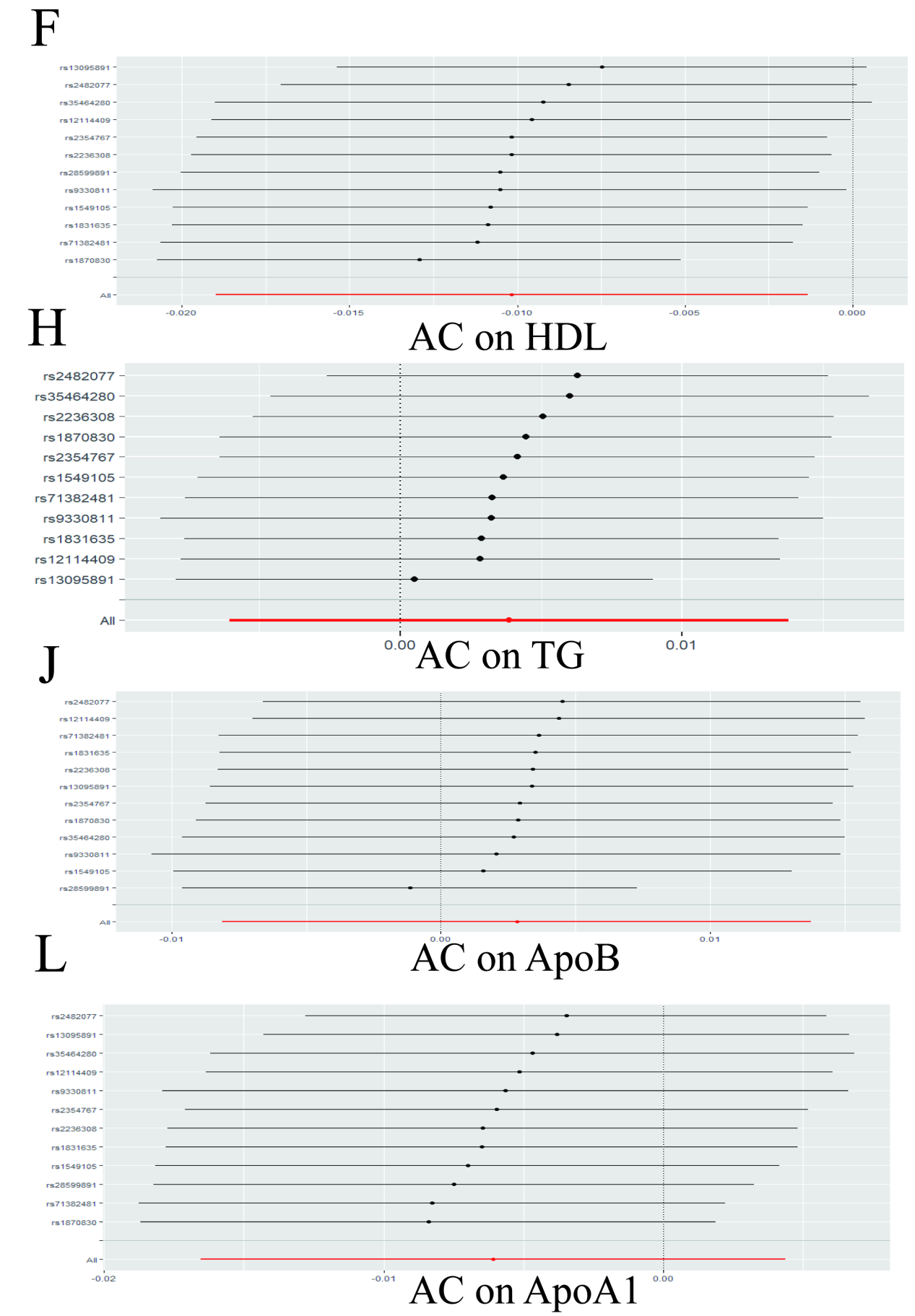

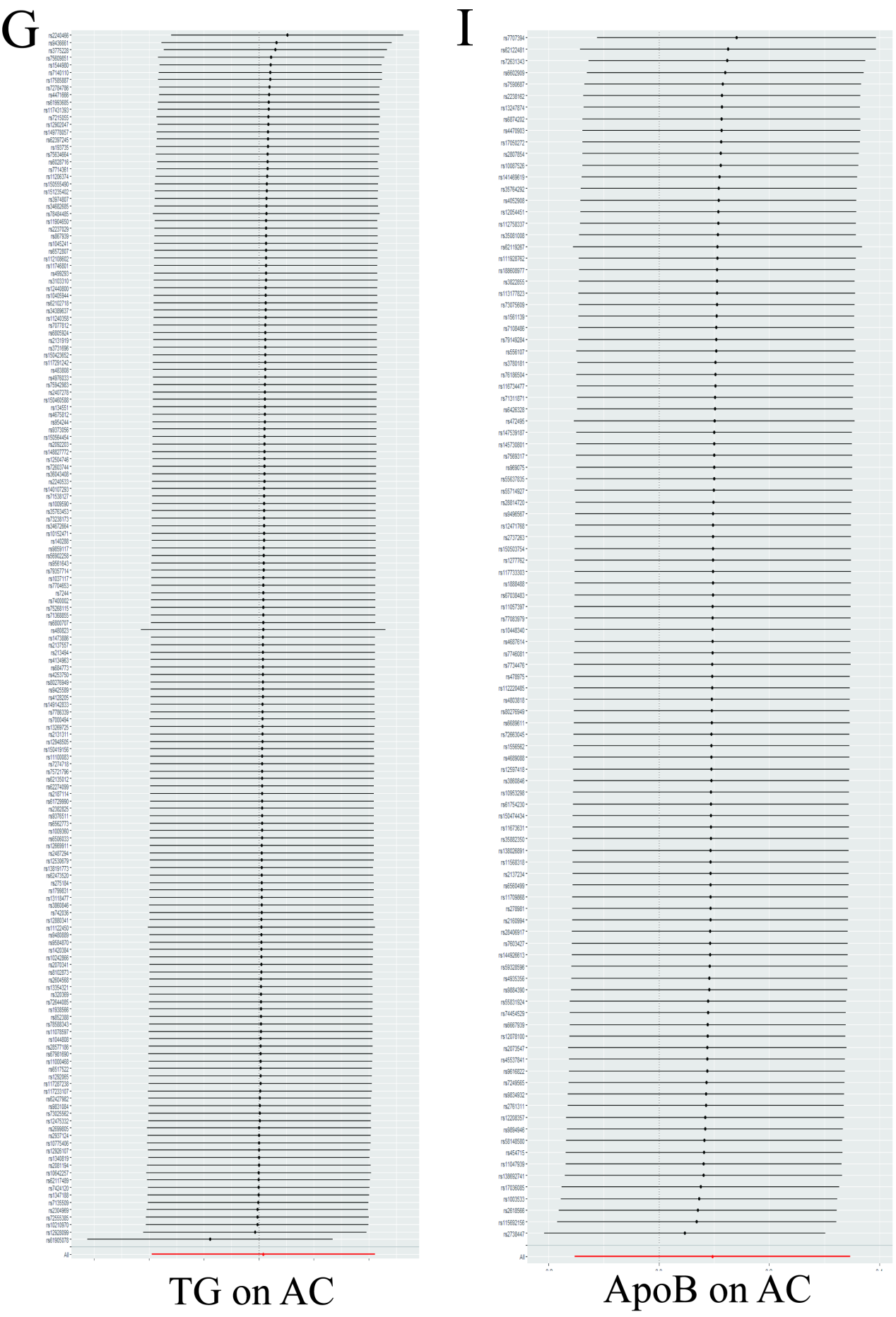


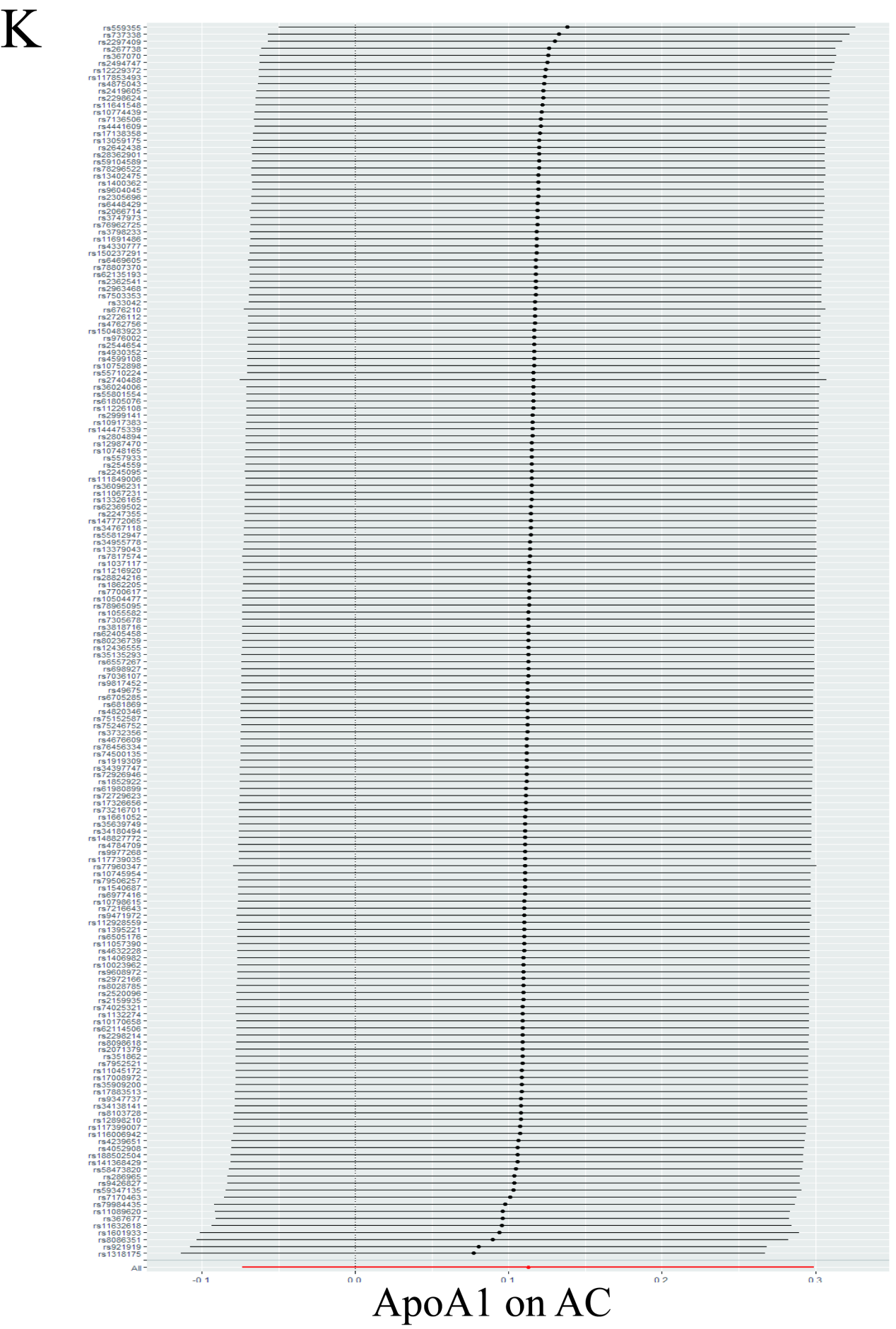


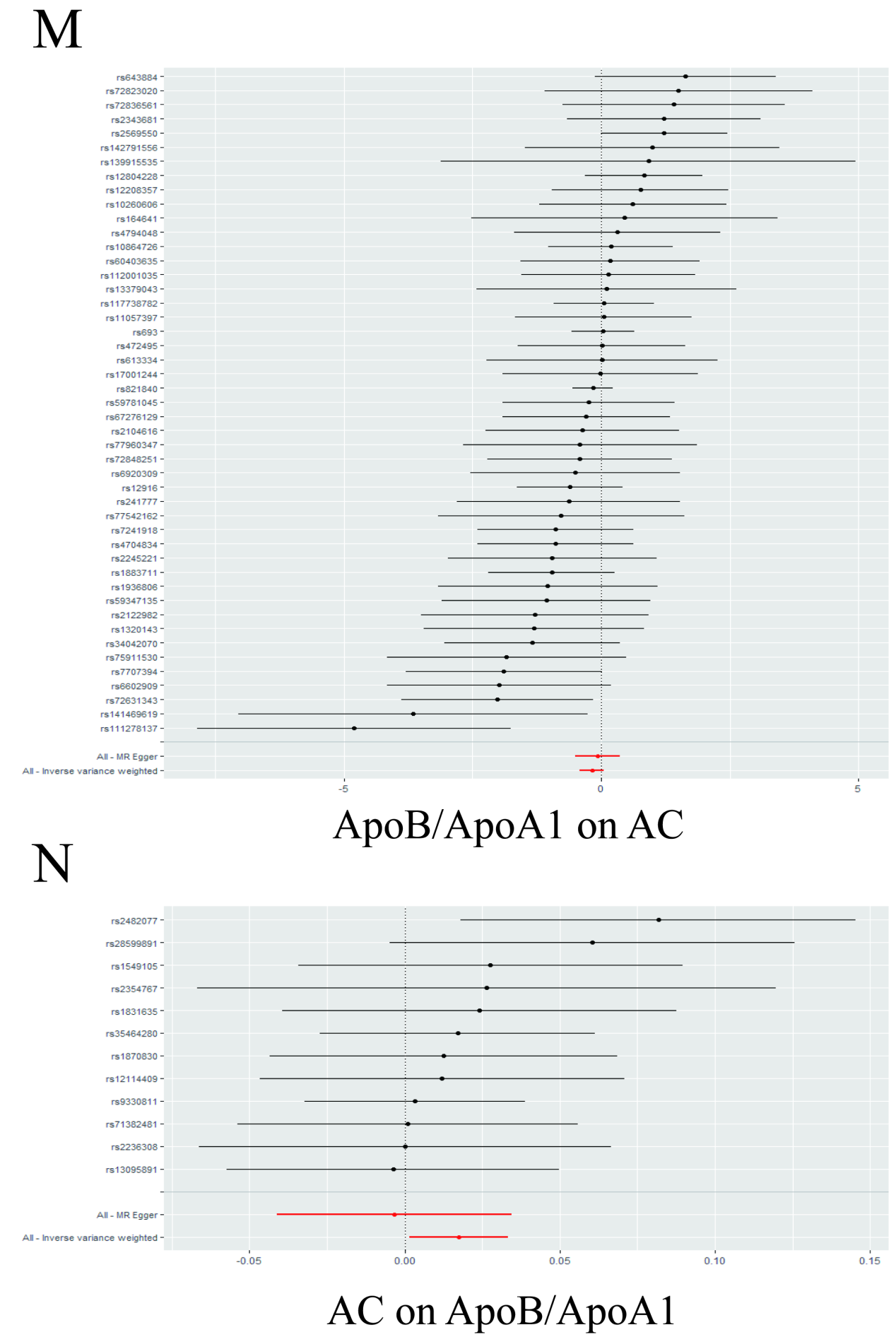


MR: mendelian randonmization; EH: essential hypertension; AC: adhesive capsulitis; LDL: low density lipoprotein; HDL: high density lipoprotein; TG: triglyceride; ApoB: apolipoprotein B; ApoA1: apolipoprotein A1; ApoB/ApoA1:apolipoprotein B/apolipoprotein A1 ratio
