# Supplementary material for: Hypertension, Dyslipidemia, and Adhesive Capsulitis: A Bidirectional Two‐Sample Mendelian Randomization Study of the European Population
Source: Genet Res (Camb). 2026 May 17;2026:6618466. doi: 10.1155/genr/6618466 (PMC13180687; doi:10.1155/genr/6618466)

**Supplementary Figure 4** Forest plot of the MR results between exposures and outcome

**The forward MR results**


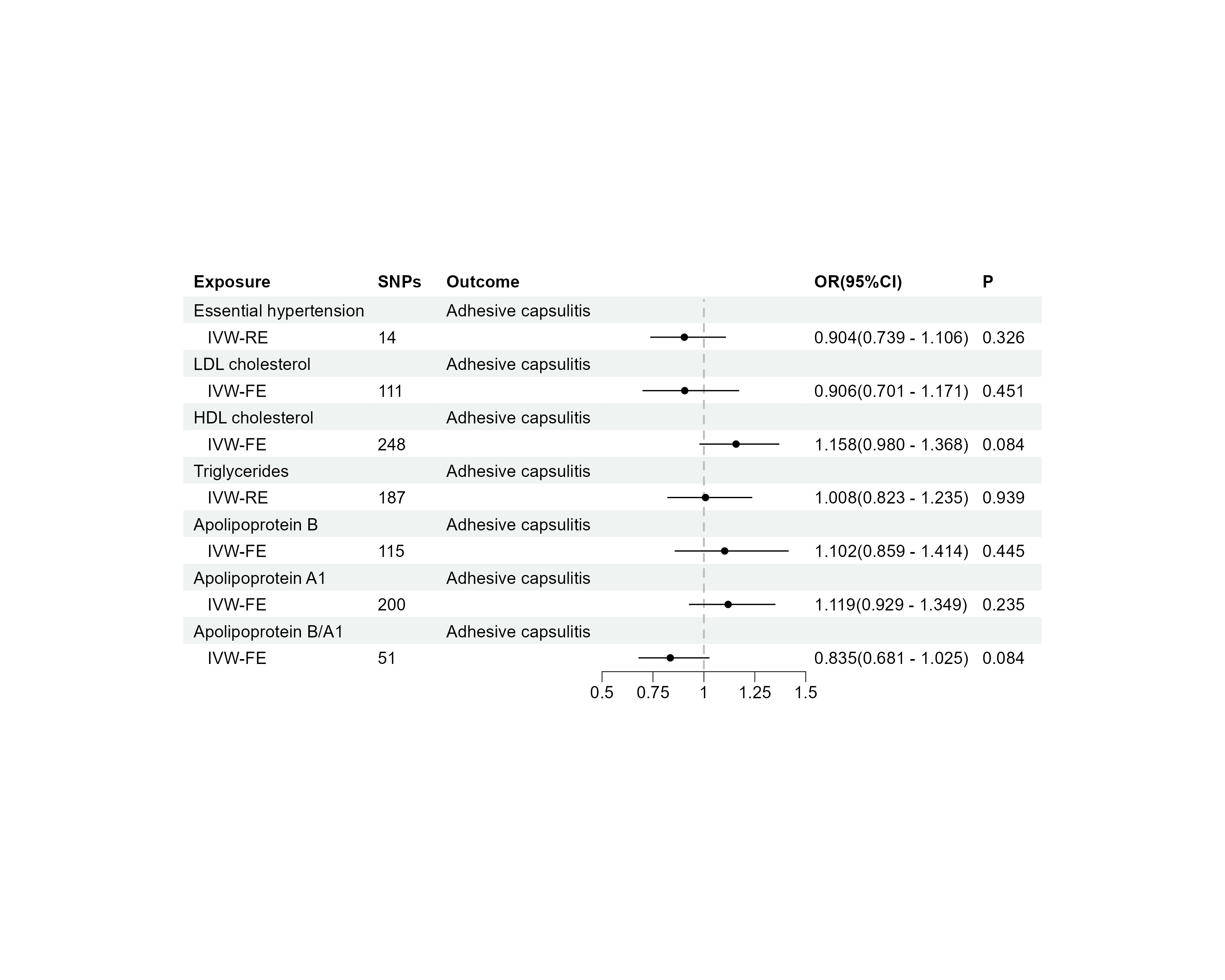


**The reverse MR results**


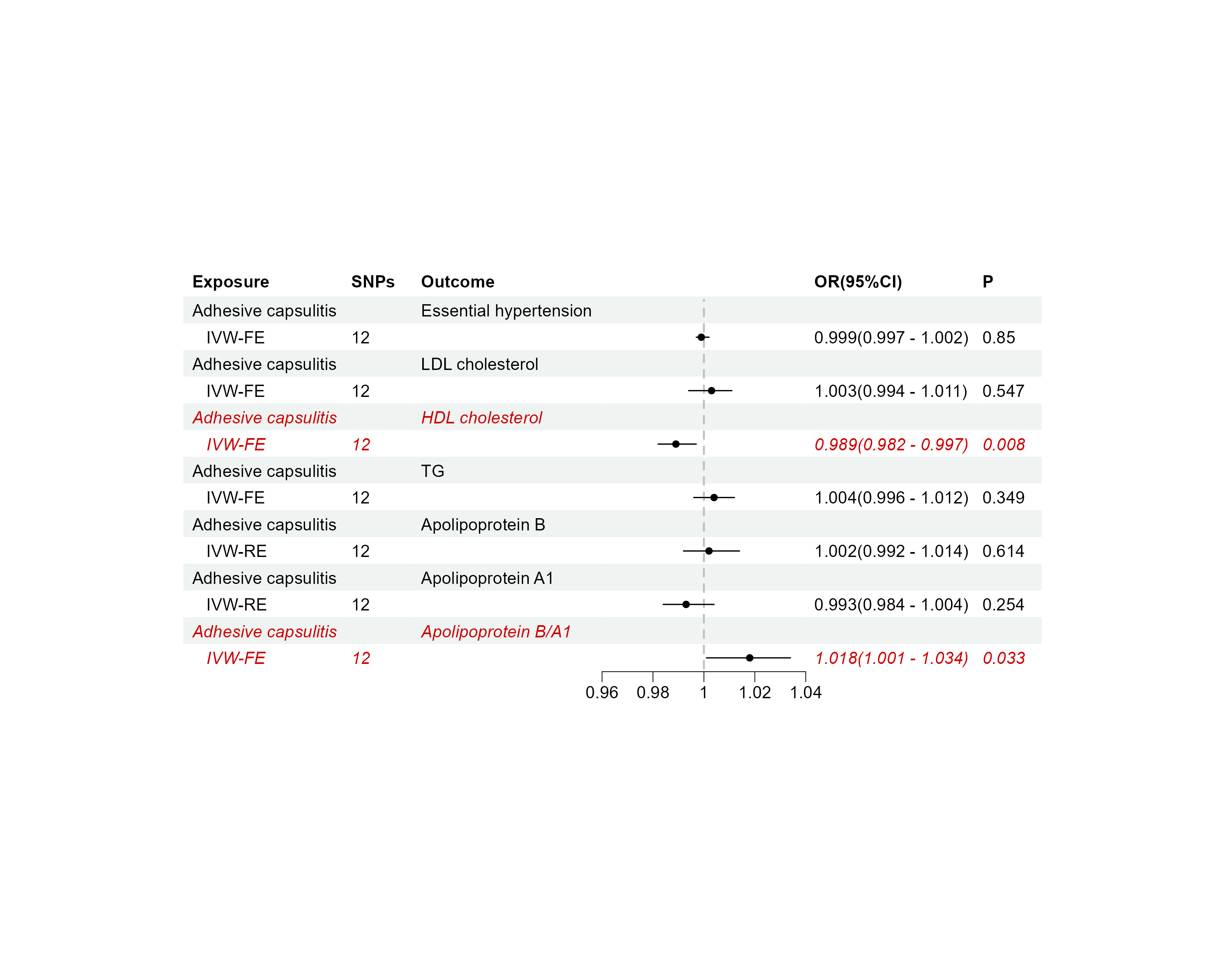

Supplement: Supplementary file 4 — Supporting Information 4 Supporting 4. Supporting Figure 4. Forest plot of the MR results between the exposures and outcome. [file GENR-2026-6618466-s011.doc]
