# Supplementary material for: Hypertension, Dyslipidemia, and Adhesive Capsulitis: A Bidirectional Two‐Sample Mendelian Randomization Study of the European Population
Source: Genet Res (Camb). 2026 May 17;2026:6618466. doi: 10.1155/genr/6618466 (PMC13180687; doi:10.1155/genr/6618466)
Supplement: Supplementary file 5 — Supporting Information 5 Supporting 5. Supporting Figure 5. The number of SNPs filtered at each step. [file GENR-2026-6618466-s010.doc]

**Supplementary Figure 5** The number of SNPs filtered at each step

p<5×10^-8^

Excluded SNPs in LD

(r^2^>0.01，kb<10,000)

Essential hypertension：40

LDL cholesterol：179

HDL cholesterol：356

Triglycerides：313

Apolipoprotein B：190

Apolipoprotein A1：300

Apolipoprotein B/A1 ratio:72

Apolipoprotein B/A1 ratio：

Essential hypertension：40

LDL cholesterol：179

HDL cholesterol：356

Triglycerides：313

Apolipoprotein B：190

Apolipoprotein A1：300

Apolipoprotein B/A1 ratio:72

Adhesive Capsulitis：12

Essential hypertension：14

LDL cholesterol：111

HDL cholesterol：248

Triglycerides：187

Apolipoprotein B：115

Apolipoprotein A1：200

Apolipoprotein B/A1 ratio:51

Adhesive Capsulitis：12

Exposure:

Essential hypertension：11

LDL cholesterol：102

HDL cholesterol：225

Triglycerides：174

Apolipoprotein B：106

Apolipoprotein A1：180

Apolipoprotein B/A1 ratio:46

Outcome:

Adhesive Capsulitis

Exposure:

Essential hypertension：11

LDL cholesterol：101

HDL cholesterol：221

Triglycerides：172

Apolipoprotein B：106

Apolipoprotein A1：178

Apolipoprotein B/A1 ratio:46

Outcome:

Adhesive Capsulitis

p<5×10^-6^

Excluded SNPs in LD

(r^2^>0.01，kb<10,000)

Adhesive Capsulitis：12

**Initial SNP screening**

**F-statistic calculation**

**PhenoScanner screening**

**Extract the outcome data**

**Harmonize data**

Exposure:

Adhesive Capsulitis

Outcome:

Essential hypertension：11

LDL cholesterol：13

HDL cholesterol：13

Triglycerides：13

Apolipoprotein B：13

Apolipoprotein A1：13

Apolipoprotein B/A1 ratio:12

Exposure:

Adhesive Capsulitis

Outcome:

Essential hypertension：11

LDL cholesterol：12

HDL cholesterol：12

Triglycerides：12

Apolipoprotein B：12

Apolipoprotein A1：12

Apolipoprotein B/A1 ratio:12
