# Supplementary figures and images for: Hypertension, Dyslipidemia, and Adhesive Capsulitis: A Bidirectional Two‐Sample Mendelian Randomization Study of the European Population
Source: Genet Res (Camb). 2026 May 17;2026:6618466. doi: 10.1155/genr/6618466 (PMC13180687; doi:10.1155/genr/6618466)

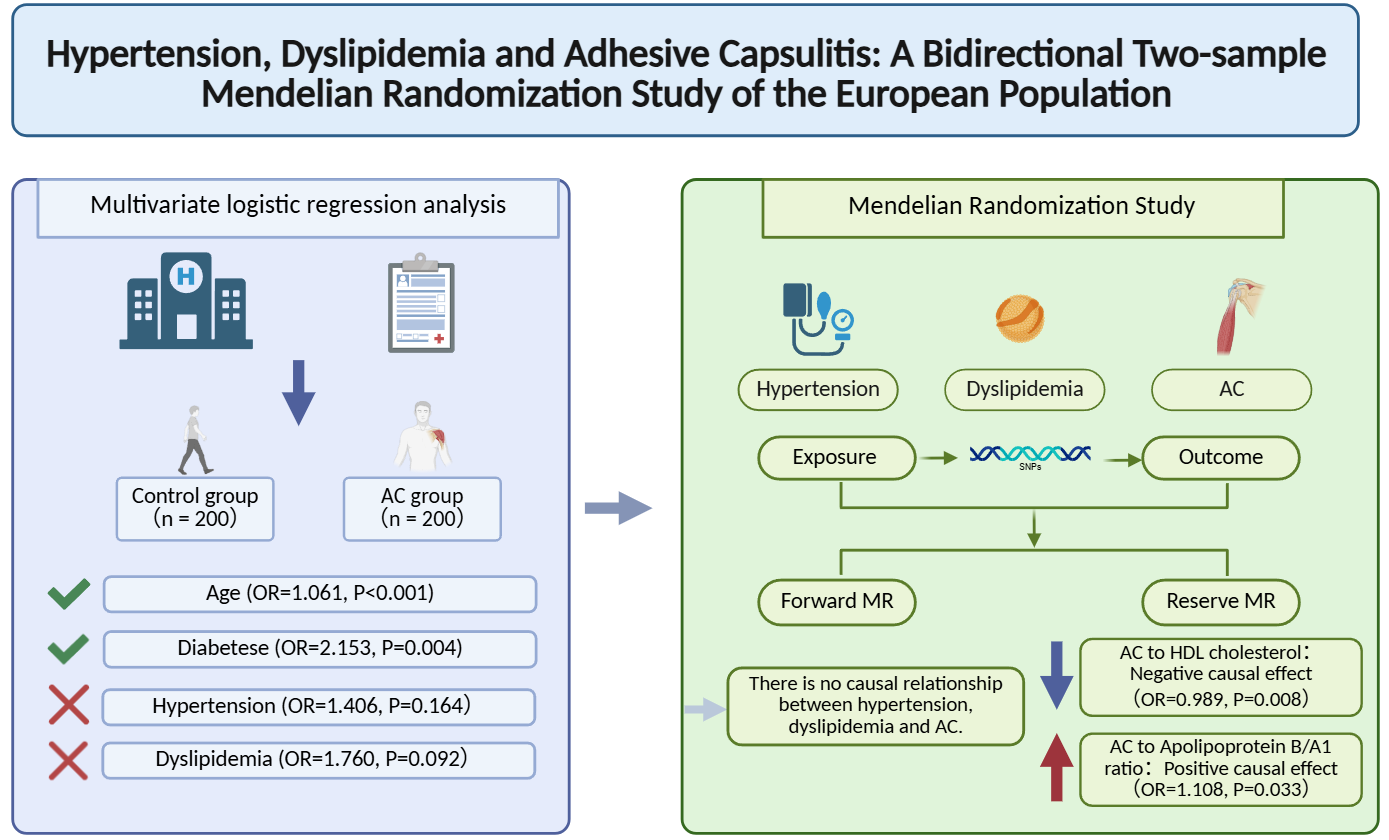

Supplement: Supplementary file 14 — Supporting Information 14 Graphical abstract: This graphical abstract summarizes a bidirectional two‐sample Mendelian randomization study investigating the relationship between hypertension, dyslipidemia, and adhesive capsulitis (AC) in a European population. Multivariable logistic regression identified age and diabetes as significant risk factors for AC, while hypertension and dyslipidemia were not significantly associated. Forward Mendelian randomization analysis further showed no causal effect of hypertension or dyslipidemia on AC, whereas reverse MR suggested that AC may have a negative causal effect on HDL cholesterol and a positive causal effect on the apolipoprotein B/A1 ratio. [file GENR-2026-6618466-s002.png]
